# MiR-21-5p in urinary extracellular vesicles is a novel biomarker of urothelial carcinoma

**DOI:** 10.18632/oncotarget.14969

**Published:** 2017-02-01

**Authors:** Kyosuke Matsuzaki, Kazutoshi Fujita, Kentaro Jingushi, Atsunari Kawashima, Takeshi Ujike, Akira Nagahara, Yuko Ueda, Go Tanigawa, Iwao Yoshioka, Koji Ueda, Rikinari Hanayama, Motohide Uemura, Yasushi Miyagawa, Kazutake Tsujikawa, Norio Nonomura

**Affiliations:** ^1^ Department of Urology, Osaka University Graduate School of Medicine, Osaka, Japan; ^2^ Laboratory of Molecular and Cellular Physiology, Osaka University Graduate School of Pharmaceutical Science, Osaka, Japan; ^3^ Department of Urology, Osaka General Medical Center, Osaka, Japan; ^4^ Department of Urology, Osaka Police Hospital, Osaka, Japan; ^5^ Cancer Proteomics Group, Genome Center, Japanese Foundation for Cancer Research, Tokyo, Japan; ^6^ Department of Immunology, Kanazawa University Graduate School of Medical Sciences, Ishikawa, Japan

**Keywords:** extracellular vesicles, urothelial carcinoma, microRNA, urine, biomarker

## Abstract

**Background:**

Extracellular vesicles are lipid bilayer vesicles containing protein, messengerRNA and microRNA. Cancer cell-derived extracellular vesicles may be diagnostic and therapeutic targets. We extracted extracellular vesicles from urine of urothelial carcinoma patients and the control group to identify cancer-specific microRNAs in urinary extracellular vesicles as new biomarkers.

**Materials and methods:**

microRNA from urinary extracellular vesicles extracted from 6 urothelial carcinoma patients and 3 healthy volunteers was analyzed. We verified candidate microRNAs in an independent cohort of 60 urinary extracellular vesicles samples. To normalize the microRNA expression level in extracellular vesicles, we examined the following in extracellular vesicles: protein concentration, CD9 intensity, amounts of whole miRNAs, RNA U6B small nuclear expression and the creatinine concentration of original urine correlating with the counts of extracted extracellular vesicles measured by the NanoSight™ system.

**RESULTS:**

From the microarray results 5 microRNAs overexpressed in urinary extracellular vesicles of urothelial carcinoma patients were identified. Creatinine concentration of original urine correlated most with particle counts of extracellular vesicles, indicating that creatinine could be a new tool for normalizing microRNA expression. MiR-21-5p was the most potent biomarker in urinary extracellular vesicles (sensitivity, 75.0%; specificity, 95.8%) and was also overexpressed in urinary extracellular vesicles from urothelial carcinoma patients with negative urine cytology. For the subgroup with negative urine cytology, the sensitivity was 75.0% and specificity was 95.8%.

**Conclusion:**

MiR-21-5p in urinary extracellular vesicles could be a new biomarker of urothelial carcinoma, especially for urothelial carcinoma patients with negative urine cytology.

## INTRODUCTION

Urothelial carcinomas (UC), including bladder cancer, are the sixth most common cancer in the United States [1]. Cancer screening and early diagnoses are primarily important in improving patient survival. The current standard of screening for UC is urine cytology, and its value in diagnosis UC is based on a sensitivity of 38.0% and a specificity of 98.3% [2]. It is of limited value owing to its poor sensitivity, especially for low-grade tumors [3–5]. Cystoscopy can offer high diagnostic accuracy, but it is costly and invasive, and in general, the urologists would perform cystoscopy only on the patients with a high level of suspicion of UC (e.g., positive urine cytology or gross hematuria). For these reasons, noninvasive and more sensitive biomarkers are needed.

Extracellular vesicles (EVs), including exosomes and microvesicles, are lipid bilayer vesicles (usually 40-200 nm in diameter) containing protein, mRNA and micro RNA (miRNA) that are secreted from most cell types and tumor cells into various bodily fluids [6, 7]. EVs derived from cancer cells promote cancer progression by communication with other cells to induce angiogenesis and cancer progression [8–11]. These vesicles have potential as both diagnostic and therapeutic targets. It is hypothesized that urine might contain abundant cancer-specific EVs derived from UC because UC directly contact urine.

MiRNA is a small noncoding RNA molecule of 20–25 nucleotides, which downregulates gene expression through translational repression or mRNA degradation.

In this study, we extracted EVs from the urine of patients with UC and the control, and focused on the miRNAs overexpressed in urinary EVs in UC patients compared to the control to identify cancer-specific miRNAs in urinary EVs for possible use as new biomarkers.

## RESULTS

### EVs from human urine

**Figure 1:**
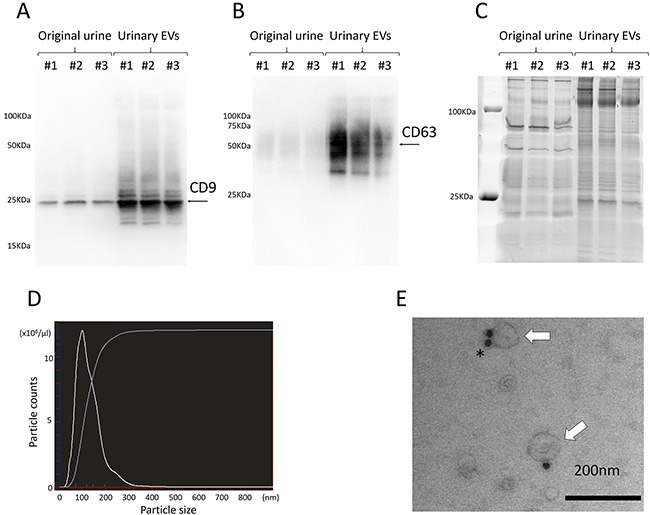
**A, B**. Western blotting showed the expression of specific proteins ((A) CD9 and (B) CD63) in urinary EVs. **C**. A representative gel stained with SYPRO Ruby, showing the protein profile of original urine and urinary EVs. **D**. Nanoparticle tracking analysis revealed that almost all of the particles extracted by ultracentrifugation were under 200 nm in size. **E**. Electron microscopy shows urinary EVs immunolabeled with anti-CD9 antibody conjugated by 20-nm protein gold nanoparticles (white arrows indicate EVs, and the asterisk indicates gold nanoparticles). Data are representative of 3 (A, B, C and D) and one (E) independent experiment.

EVs were extracted from 38.5-ml samples of voided urine by differential centrifugation and were confirmed by Western blot using the well-defined EV markers CD9 and CD63 (Figure 1A–1C). NanoSight^™^ particle tracking analysis showed that the particles extracted by ultracentrifugation were almost all under 200 nm in size (Figure 1D). Electron microscopic analysis of EVs from human urine revealed rounded membrane-bound vesicles under 200 nm in size that expressed CD9 on their surface (Figure 1E).

### MiRNAs microarray analysis of urinary EVs

We performed microarray analysis of miRNA of urinary EVs from 6 UC patients and 3 healthy volunteers (HV) and identified miRNAs that expressed more highly in urinary EVs in UC patients compared to those in HV (Supplementary Figure 1). Table 1 showed the patient characteristics. The coefficient of variation on the microarray was 204.33. We selected 5 miRNAs that showed a more than 2.5-fold higher expression and p-value <0.1 in urinary EVs of UC patients compared to those of HV (Table 2).

**Table 1:** Patient characteristics for miRNA microarray

	UC patients (n=6)		Control (n=3)
Age (years) [median (range)]	67.5 (56-83)		53 (41-55)
Gender (male/ female)	6 / 0		2 / 1
T stage	Ta	2	
	T1	1	
	T3	2	
	Tis	1	
Pathological grade	Low grade	2	
	High grade	4	
UC : urothelial carcinoma			

**Table 2:** Five candidate miRNAs from the miRNA microarray analysis of urinary EVs of UC patients and Control

	Fold change	p-value
miR-155-5p	28.792	0.002
miR-15a-5p	3.889	0.031
miR-21-5p	3.756	0.089
miR-132-3p	2.878	0.079
miR-31-5p	2.654	0.022

EV, extracellular vesicles; UC, urothelial carcinoma.

### Normalization of miRNAs in urinary EVs

**Figure 2:**
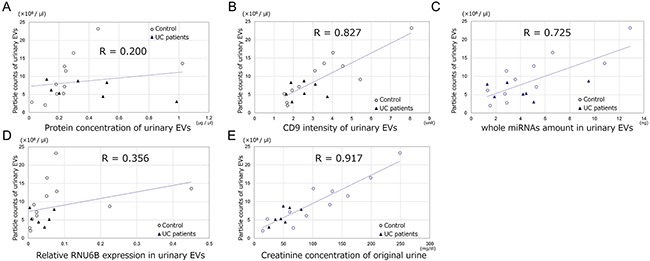
**The association between the particle counts of urinary EVs and A.** protein concentration of urinary EVs, **B**. CD9 intensity of urinary EVs, **C**. amounts of whole miRNAs in EVs, **D**. RNU6B expression in urinary EVs and **E**. the creatinine concentration of original urine.

The creatinine concentration of original urine most correlated with the particle counts of extracted urinary EVs (r=0.917, p<0.0001) when compared to the protein concentration (r=0.200, p=0.425), CD9 intensity (r=0.827, p<0.0001) of EVs and amounts of whole miRNAs (r=0.725, p<0.0001) and RNA U6B small nuclear (RNU6B) expression (r=0.356, p=0.145) in EVs (Figure 2A–2E). The creatinine concentration of original urine correlated most with the particle counts of extracted urinary EVs (r=0.916, p<0.0001) only in the control group when compared to the protein concentration (r=0.536, p=0.088), CD9 intensity (r=0.879, p=0.0004) of EVs, amounts of whole miRNAs (r=0.844, p=0.0010) and RNU6B expression (r=0.359, p=0.275) in EVs. We thus decided to use the creatinine concentration of original urine for the normalization of miRNAs in urinary EVs. There was no significant difference in the counts of EVs between UC patients and the control group (p=0.189) or between males and females (p=0.153).

### MiRNA expression in the validation cohort

The validation cohort comprised 60 urinary EVs samples (36 samples from UC patients and 24 samples from the control group without UC). UC samples comprised 18 non-invasive UC and 18 invasive UC, and the control group without UC comprised 11 donors for kidney transplantation, 5 healthy persons in our departments and 8 postoperative patients of UC with no evidence of disease after the surgery. The patient characteristics are shown in Table 3.

**Table 3:** Patients characteristics in the validation cohort

	ControlN=24	UC patientsN=36	p-value
Age (years) [median (range)]	70 (34-81)	70.5 (47-91)	<0.0001
Gender n, (%)			
Male	17 (70.8)	29 (80.6)	0.386
Female	7 (29.2)	7 (19.4)	
Urine cytology n, (%)			
Negative	24 (100)	20 (55.6)	<0.0001
Positive	0 (0)	16 (44.4)	
Creatinine concentration of original urine (mg/dl) [median (range)]	64.6(10.8-199.1)	76.6(17.6-261.8)	0.446
UC pathological stage n, (%)			
Ta	—	18 (50.0)	
T1	—	9 (25.0)	
≧T2	—	9 (25.0)	
UC pathological grade n, (%)			
Low grade	—	9 (25.0)	
High grade	—	27 (75.0)	
UC, urothelial carcinoma.			

**Figure 3:**
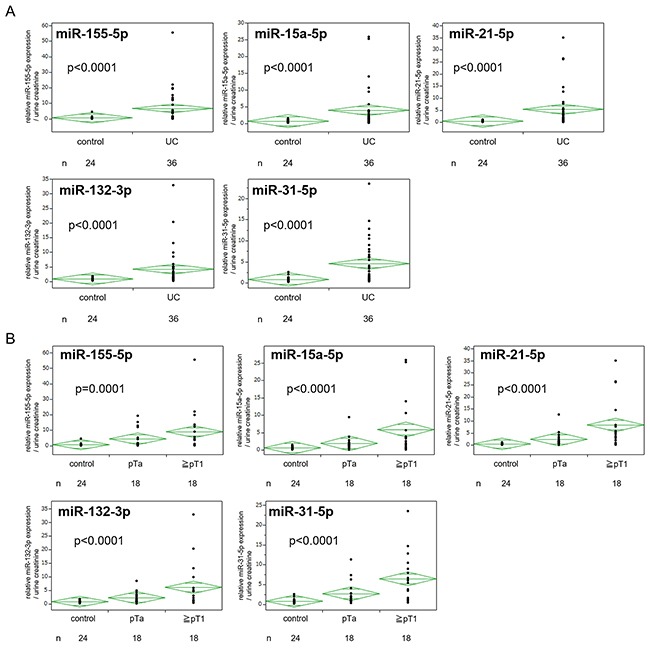
**A**. Expression of five candidate miRNAs in urinary extracellular vesicles (EVs) in the validation cohort. **B**. Expression of five candidate miRNAs in urinary EVs between cancer stages. UC, urothelial carcinoma.

**Figure 4:**
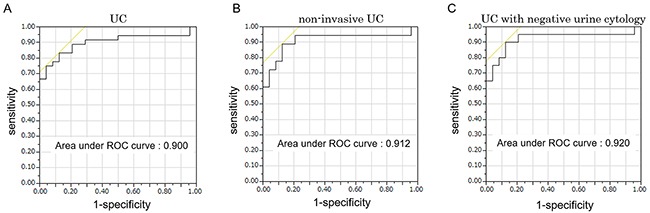
**A**. ROC curve for the classifier of UC using miR-21-5p in urinary extracellular vesicles (EVs). **B**. ROC curve for the classifier of non-invasive UC using miR-21-5p in urinary EVs. **C**. ROC curve for the classifier of UC in negative urine cytology subgroup using miR-21-5p in urinary EVs.

Five candidate miRNAs (miR-155-5p, miR-15a-5p, miR-21-5p, miR-132-3p and miR-31-5p) were all significantly expressed more highly in urinary EVs of UC patients compared to those of the control (all, p<0.0001) (Figure 3A). There was no significant difference in the expression of miRNAs between males and females (p=0.310, 0.662, 0.700, 0.319 and 0.234, respectively). Pathological stage was also associated with the expression levels of these miRNAs (Figure 3B). The five miRNAs all correlated significantly with each other (Spearman's r=0.741- 0.928; all, p<0.001). For the ability to detect UC, miR-21-5p in urinary EVs was the most potent biomarker, and the area under the receiver-operator characteristics (ROC) curve (AUC) was 0.900 (95% CI 0.782-0.958, p<0.0001, Figure 4A). The sensitivity and specificity of the model at the best cutoff value were 75.0% and 95.8%, respectively, and this result was better than that of urine cytology in this cohort (sensitivity of 44.4% and specificity of 100%, Youden's index: 0.708 vs 0.444). Supplementary Figure 2 showed ROC curve using other 4 miRNAs. For the subgroup of patients with non-invasive UC, miR-21-5p resulted in a sensitivity of 72.2% and specificity of 95.8% using the cutoff value (AUC=0.912, 95% CI 0.725-0.976, p<0.0001, Figure 4B). MiR-21-5p was also expressed more highly in urinary EVs in UC patients with negative urine cytology, and for the subgroup with negative urine cytology, the sensitivity was 75.0% and specificity was 95.8% using the cutoff value (AUC=0.920, 95% CI 0.748-0.978, p<0.0001, Figure 4C). These results indicated that miR-21-5p in urinary EVs could potentially be a biomarker to detect the early stage and negative urine cytology of UC. We compared the expression level of miR-21-5p in EVs before the treatment with those after the treatment in 12 matched-pair samples, and the expression level of miR-21-5p significantly decreased postoperatively (p=0.0362). There was no significant correlation between the recurrence-free survival of patients with UC and the expression level of miR-21-5p in EVs (above the cutoff value vs below, p=0.482). We also examined EVs from carcinoma *in situ* (CIS, n=3), and miR-21-5p was significantly overexpressed in urinary EVs from CIS compared to the control (p=0.0151).

Next, we examined the cases of pyuria and hematuria. The expression level of miR-21-5p in urinary EVs was higher than the cutoff value in all cases with pyuria from acute cystitis (n=5), but in the validation cohort excluding the cases with WBCs in the urinalysis (16 cases from UC) and the cases without urinalysis data (7 cases from UC and 4 cases from the control), the expression level of miR-21-5p was also significantly higher in urinary EVs of UC patients compared to that of the control (p=0.0009). The sensitivity and specificity were 61.5% and 100% in the subgroup, respectively. The expression level of miR-21-5p in urinary EVs was lower than the cutoff value in all cases with benign hematuria (n=5). In addition, we examined the blood spike in artificial hematuria to assess the effect of blood contamination in urine (n=3). The expression level of miR-21-5p in EVs extracted from artificial hematuria (Ct value (median±S.D.) =25.66±0.86) did not vary compared to that of the pre-spiked urine (26.08±0.98, p=0.275). These results also indicated that the expression level of miR-21-5p in EVs was independent of blood contamination in urine.

Finally, we performed univariate logistic regression analysis, which showed that age, urine cytology and all five candidate miRNAs in EVs were associated with the presence of UC. Multivariate logistic regression analysis revealed that after adjustment for age, gender and urine cytology, miR-21-5p in EVs was a significant predictor of the presence of UC (Table 4, multivariate model 1). In addition, we performed stepwise multiple logistic regression analysis, which showed that age, miR-21-5p and miR-132-3p were significant predictors of the presence of UC (Table 4, multivariate model 2). The AUC for the prediction of this model was 0.950, but it was not significantly different from the model using only miR-21-5p in urinary EVs (AUC=0.900, p=0.350).

**Table 4:** Logistic regression analysis of variables associated with the presence of UC

Variable included	Univariate			Multivariate model 1			Multivariate model 2		
	Odds ratio	95% CI	p-value	Odds ratio	95% CI	p-value	Odds ratio	95% CI	p-value
Age	1.105	1.050-1.180	<0.0001	1.044	0.969-1.155	0.273	1.106	1.014-1.253	0.018
Gender (M/F)	1.705	0.503-5.817	0.386	5.455	0.515-118.2	0.168			
Urine cytology	1.20×10^9^	—	<0.0001	2.332×10^9^	4.807-Inf	0.003			
(P vs N)									
Urinary EVs									
miR-155-5p	2.124	1.308-4.924	<0.0001						
miR-15a-5p	8.972	2.765-44.15	<0.0001						
miR-21-5p	27.35	4.520-360.7	<0.0001	65.62	4.922-3820	<0.0001	2864	22.14-3.506×10^6^	<0.0001
miR-132-3p	5.641	2.017-26.02	<0.0001				0.006	3.719×10^−5^-0.299	0.0076
miR-31-5p	3.956	1.721-14.467	<0.0001						

EVs: extracellular vesicles

### MiR-21-5p in urothelial carcinoma

After 72 h incubation, the human bladder cancer cell lines (UMUC3, 5637, J82 and RT4) secreted more EVs than the immortalized urothelial cell line (SV-HUC-1). The expression level of miR-21-5p in EVs from the cancer cell lines was higher than that from SV-HUC-1 (Supplementary Figure 3).

From the results of microarray analysis of UC tissue specimens, miR-21-5p was 2.6-fold more highly expressed in UC tissues than in normal tissues (p=0.009)

## DISCUSSION

EVs include exosomes and microvesicles. In general, exosomes are formed by budding from late endosomes, are 40-200 nm in diameter, and change protein expressions of nearby cells or distant organs according to the proteins, mRNA and miRNAs they carry. In contrast, microvesicles are on the scale of 100-1000 nm in diameter and are produced through plasma membrane budding with lipids and proteins that reflect their cellular membrane to target designated cells [7, 12]. However, the strict separation of these vesicle types by size or biogenesis has not been established [13]. In this study we used the term extracellular vesicles.

Because the concentration of EVs secreted from kidney, urothelial epithelium and UC are altered by urine volume, EVs should somehow be normalized. In several studies, RNU6B was used as an endogenous control to normalize the expression data of miRNA in clinical tissue specimens. However, for normalization of miRNA of EVs, especially in urine, several studies have reported some miRNAs to be internal controls, but no consensus has been reached yet [14–16]. In our study, to use the particle counts of EVs for normalization of the miRNA expression in EVs, we analyzed the factors correlating with the counts of urinary EVs. As a result, the creatinine concentration of original urine correlated most with the counts of urinary EVs, and there was no difference in the particle counts in urinary EVs between UC patients and the control group. From this result, we speculated that almost all urinary EVs were derived from kidney and normal urothelial epithelium, and that only a very small population of EVs were derived from UC. Although the counts of EVs derived from UC was small, the cancer-specific miRNAs in EVs were abundant compared to those from kidney and normal urothelial epithelium. The protein concentration of urinary EVs did not correlate with the counts of EVs probably because urine contains various proteins such as the Tamm–Horsfall protein and abundant protein in EVs from UC. The CD9 intensity in urinary EVs correlated moderately with the counts of EVs, but the reason it was inferior to the creatinine concentration might be due to the presence of CD9-negative or sparse EVs. RNU6B expression in EVs did not correlate with the counts of EVs probably because urinary EVs might not contain RNU6B equally per each particle. Some studies used creatinine concentration for the normalization of proteins in urinary EVs [17–19], and our study also showed that creatinine concentration could be used for the normalization of miRNA expression in EVs for UC.

Although several studies have reported on miRNA in the urine of UC patients, there are few studies on miRNA in urinary EVs of UC patients [20–22]. Armstrong et al. [20] reported the miRNA microarray analysis of matched formalin-fixed, paraffin-embedded (FFPE)-tumor tissue, plasma, urinary EVs and WBCs from patients with bladder cancer and showed the overexpression of miR-4454 and miR-21 in FFPE-tumor tissue, urinary EVs and WBCs. However, they did not validate actual expression levels of the miRNAs by using RT-PCR-based testing in a larger number of clinical samples. Long et al. [21] reported that the expression patterns varied between miRNA in urinary EVs and that in the supernatant of urine and showed the outcome of cancer detection using four cell-free miRNAs (miR-26a, miR-93, miR-191 and miR-940). To our knowledge, our study is the first report to evaluate miRNAs in urinary EVs as new biomarkers of UC by quantitative analysis of RT-PCR.

MiR-21-5p has been reported to be overexpressed in multiple human solid tumors, including UC [23–26]. The function of miR-21-5p in cancer tissue was reported to promote epithelial-mesenchymal transition, to enhance cell motility and proliferation, and to inhibit apoptosis [27]. Several studies showed that the overexpression of miR-21 is associated with a poorer prognosis in bladder cancer [28]. These data suggest that miR-21-5p was an oncomiR, and miR-21-5p-abundant cancer cells might release EVs containing miR-21-5p to other cells such as normal cells and cancer cells with low miR-21-5p expression. MiR-21-5p in urinary EVs was reported to be up-regulated with prostate cancer [29], and there were also some reports on miR-21-5p in EVs of other biofluids for various cancers, but the function of miR-21-5p in EVs remains unproven [30, 31]. Further study is necessary to elucidate the functional role of this miRNA in EVs.

This study has several limitations. Because it is a pilot study with a small population size, further large-scale and multi-institutional studies are warranted to confirm our findings. The expression of miR-21-5p in urinary EVs was also higher in cases of acute cystitis. However some tumor markers, such as prostate-specific antigen (PSA), would be increased in the case of the inflammation. In the validation cohort excluding the cases with WBCs in the urinalysis, the expression of miR-21-5p was still significantly higher in urinary EVs of UC patients. From these results, miR-21-5p in urinary EVs would be useful to detect UC in combination with urinalysis.

In conclusion, our study suggested that the creatinine concentration of original urine, which is easily measurable, could be a tool for the normalization of urinary EVs, and miR-21-5p in urinary EVs could be a new tumor biomarker of UC, especially for UC patients with negative urine cytology. Further large-scale and multi-institutional studies are necessary to validate these initial findings.

## MATERIALs AND METHODS

### Patients and the control group

The study protocol was reviewed and approved by the appropriate institutional ethics committees. Approval and written informed consents were obtained from our Institutional Review Board and patients, respectively, before initiating the study. Urine samples were collected from patients who were histologically diagnosed as having urothelial carcinoma and the control group from 2013 through 2016 at Osaka University Hospital, Osaka General Medical Center and Osaka Police Hospital. The control group included donors for kidney transplantation, healthy volunteers and postoperative patients of UC. Healthy volunteers were defined as those without a medical history of UC. Postoperative patients of UC were defined as patients with no evidence of disease recurrence after the surgery. Samples of pyuria and benign hematuria were collected from the patients with urological diseases other than UC (acute cystitis, urinary tract stone) and the patients after urological surgery resulting in hematuria. Urine samples from UC patients and the donors for kidney transplantation were collected between admission and the surgery. Other samples, including those for pyuria and benign hematuria, were collected in our outpatient clinic. We collected urine samples consecutively without the limitation as to the tumor size.

Histological diagnosis was determined on the basis of standard hematoxylin and eosin-stained sections by experienced senior pathologists. Patients were staged according to the 7th AJCC TNM staging system, and tumors were graded according to the World Health Organization 2004 criteria. The urine cytology was also evaluated by specialists according to our strict institutional criteria, in which negative urine cytology is defined to be no more than class III, and positive urine cytology is defined to be class IV and V.

### Preparation of urine and extraction of miRNA of urinary EVs

EVs were isolated from 38.5 ml of voided urine samples by differential centrifugation. Urine samples were centrifuged at 2000 ×g for 30 min to remove contaminating cells. The supernatant then was ultracentrifuged at 17,000 ×g for 30 min to pellet dead cells and salts. The supernatant was further ultracentrifuged at 130,000 ×g for 90 min. The pellets were washed with PBS, and ultracentrifuged at 130,000 ×g for 90 min. The final pellet was resuspended in 250 μl PBS and stored at -80°C for subsequent applications.

To create artificial gross hematuria, we spiked 70 μl whole blood into each 38.5-ml sample of voided urine of HV to examine the effect of blood contamination.

The protein concentration of EVs was determined using DC protein assay kit (BIO-RAD, Hercules, CA, USA). CD9 intensity of EVs was measured by CD9 sandwich ELISA as described later. MiRNA was isolated using the miRNeasy Mini Kit (Qiagen, Venlo, Netherlands), RNA MS2 (Roche Diagnostics, Mannheim, Germany) and Clean-up Kit (Qiagen) according to the manufacturer's protocol.

### Western blotting

The identity of urinary EVs had been confirmed by the presence of the specific surface proteins CD9 and CD63. Briefly, 20 μL per well of original urine and urinary EVs was separated by sodium dodecyl sulfate (SDS)-polyacrylamide gel electrophoresis (PAGE). The gels transferred to a polyvinylidene difluoride (PVDF: Thermo Fisher Scientific, Waltham, MA, USA) membrane by using the semidry transfer system (Thermo Fisher Scientific). The gels were stained with SYPRO Ruby Protein Gel Stain (Thermo Fisher Scientific). The membranes were probed with specific antibodies as indicated and then incubated with horseradish peroxidase (HRP)-conjugated antibody against mouse immunoglobulin (1:1000, Cell Signaling Technology: CST, Beverly, MA, USA), followed by detection with enhanced chemiluminescence (ECL) Western blotting detection reagents (Nacalai Tesque, Kyoto, Japan). ChemiDoc XRS Plus system (BIO-RAD) was used as a chemiluminescence detector. The following antibodies were used for immunological analysis in this study: CD9 (1:1000, 12A12, Shionogi, Osaka, Japan) and CD63 (1:1000, ab59479, Abcam, Cambridge, UK).

### NanoSight^™^ particle tracking analysis

The size and concentration of the isolated EVs were analyzed using the NanoSight^™^ particle tracking system. Each sample of EVs was diluted in EV-free pre-filtered PBS to obtain a measurable concentration between 0.5×10^8^ and 1×10^9^ particles/ml. NanoSight^™^ tracking analysis (NTA) software version 2.3 analyzed the samples at a constant temperature (25°C). All measurements were performed under identical processing conditions (NTA 2.3 build 0034, Detection Threshold: 5 Multi, Min Track Length: Auto, Min Expected Size: Auto). The counts of particles were defined to be the average counts of five fields of view.

### Transmission electron microscopy (TEM)

Samples of EVs (10 μg) were adsorbed onto a formvar/carbon-coated nickel grid for 1 h. EVs were fixed with 2% paraformaldehyde and then reacted with the first antibody (anti-CD9 antibody). Immunoreactive EVs were visualized with the second antibody preabsorbed with 20-nm gold particles (anti-mouse IgG antibody). The samples were negatively stained with 2% aqueous uranyl acetate for 15 min and observed with a JEM-1400Plus TEM (JEOL Ltd., Tokyo, Japan).

### Microarray analysis for miRNAs

MiRNA analysis of EVs was conducted using urinary EVs from 6 UC patients and 3 HV on miRNA microarray 2.0 (Affymetrix, Santa Clara, CA, USA). MiRNA analysis of UC tissue specimens was conducted using 11 matched-pair samples (tumor and normal specimens) plus 10 tumor specimens on miRNA microarray 2.0 (Affymetrix) [32]. The arrays were scanned using the Affymetrix Gene Chip Scanner 3000, and the scanned data were processed with Agilent GeneSpring GX software (Agilent).

### CD9 sandwich ELISA

Ninety-six well-plates were coated with anti-human CD9 antibodies (#156-820, Ancell Corporation, Bayport, MN, USA) and incubated for overnight at 4°C. After 5% BSA was added, EVs purified from urine were added and incubated for 3h at room temperature. Biotinylated anti-human CD9 antibodies (#156-030, Ancell Corporation) were added and incubated for 1h at room temperature. The plate was incubated for 1h at room temperature with HRP-conjugated streptavidin (#SA-5014, Vector Laboratories, Burlingame, CA, USA). The reaction was developed with 1-Step Ultra TMB-ELISA Substrate Solution (#34028, Thermo Fisher Scientific, Waltham, MA, USA). The reaction was arrested with 2N hydrochloric acid, and optical densities were recorded at 450 nm.

### Normalization of miRNA expression level in urinary EVs

To find the factor for normalization of the miRNA expression level in urinary EVs, we analyzed the factors correlated with the counts of extracted EVs. We examined the counts of particles from 18 samples of urinary EVs (7 UC patients, 6 HV and 5 post-UC patients) using NanoSight™ particle tracking analysis and analyzed the correlation of the particle counts of EVs with the protein concentration of EVs, CD9 intensity of EVs, amounts of whole miRNAs in EVs, RNU6B expression level in EVs and the creatinine concentration of the original urine. The control group included 9 males and 2 females, and UC patients included 4 males and 3 females.

### Real-time PCR (RT-PCR)

Expression of miRNAs was measured using quantitative RT-PCR (qRT-PCR). MiRNAs from urinary EVs were eluted in 14 μl RNAase-free water, and we used 3.75 μl for synthesis of 100 μl cDNA using the Mir-X miRNA First-Strand Synthesis Kit (Clontech, Mountain View, CA, USA). The qualities of miRNAs were assessed with an Agilent 2100 Bioanalyzer (Agilent Technologies, Santa Clara, CA, USA). miRBase 21 was used for miRNA mapping. For real-time PCR, 1.6 μl cDNA, 10 μl SsoAdvanced Universal SYBR Green Supermix (BIO-RAD), 0.5 μl forward primer (10 μM), 0.5 μl reverse primer (10 μM) and 7.4 μl dH_2_O were mixed to make a 20 μl reaction volume. qRT-PCR was performed with a Thermal Cycler CFX Connect (BIO-RAD). Thermal cycling conditions included an initial step at 98°C for 1 min, and 40 cycles at 95°C for 5 s and at 62-68°C for 15 s for each miRNA-specific primer and the dissociation curves and melting temperatures were recorded. PCR reactions for each sample were carried out in triplicate. As mentioned, we used the creatinine concentration of the original urine samples for normalization because it correlated most with the particle counts of extracted EVs. The levels of miRNA expression were calculated based on the standard curve, and relative expression was defined to be the ratio to the optical cut-off value determined from the ROC curve by using the Youden's index.

### EVs extraction from cell culture conditioned medium

Human bladder cancer cell lines UMUC3, J82, RT4 and immortalized urothelial cell line SV-HUC-1 were purchased from the American Type Culture Collection (Manassas, VA, USA), and bladder cancer cell line 5637 was kindly provided from the Cell Resource Center for Biomedical Research, Institute of Development, Aging and Cancer, Tohoku University. UMUC3 was cultured in Dulbecco's modified Eagle's medium (DMEM; Wako, Osaka, Japan), and 5637 and RT4 were cultured in RPMI 1640 medium (Wako). J82 was cultured in Eagle's minimum essential medium (EMEM; Wako), and SV-HUC-1 was cultured in Ham's F-12 (Wako), with 10% exosome-depleted fetal bovine serum (Thermo Fisher Scientific) and 5 μg/ml gentamaicin (MSD, NJ, USA) and 60 μg/ml tylosin (Sigma-Aldrich, St. Louis, USA), and incubated at 37°C in a humidified atmosphere containing 5% CO_2_. Cells were grown to confluence for 72 h. Conditioned media were pooled and centrifuged to obtain EVs according to the same protocol as the urine samples.

### Statistical analysis

Statistical analyses were preformed using JMP^®^ Pro 12.2.0 (SAS Institute Inc, NC, USA). The patient characteristics were compared using the Mann-Whitney U test and χ^2^-test. Univariate analysis was performed with the Mann-Whitney U test. Multiple regression analyses were performed to assess the relative contributions of factors (age, gender and urine cytology) and the candidate miRNAs in EVs. Correlation analysis was performed using Spearman analysis. Significant predictors of urothelial carcinoma were identified by logistic regression analysis. Differences were considered statistically significant when the p value was less than 0.05. The optical cutoff value for each miRNAs was determined from the ROC curve by using the Youden's index, and the sensitivity and specificity to detect UC using each miRNA were calculated according to each optical cutoff value.

## SUPPLEMENTARY MATERIALS FIGURES AND TABLES


