# Supplementary material for: MiR-21-5p in urinary extracellular vesicles is a novel biomarker of urothelial carcinoma
Source: Oncotarget. 2017 Feb 1;8(15):24668–78. doi: 10.18632/oncotarget.14969 (PMC5421878; doi:10.18632/oncotarget.14969)
Supplement: Supplementary file 1 [file oncotarget-08-24668-s001.pdf]

MiR-21-5p in urinary extracellular vesicles is a novel biomarker of urothelial carcinoma

SUPPLEMENTARY FIGURES

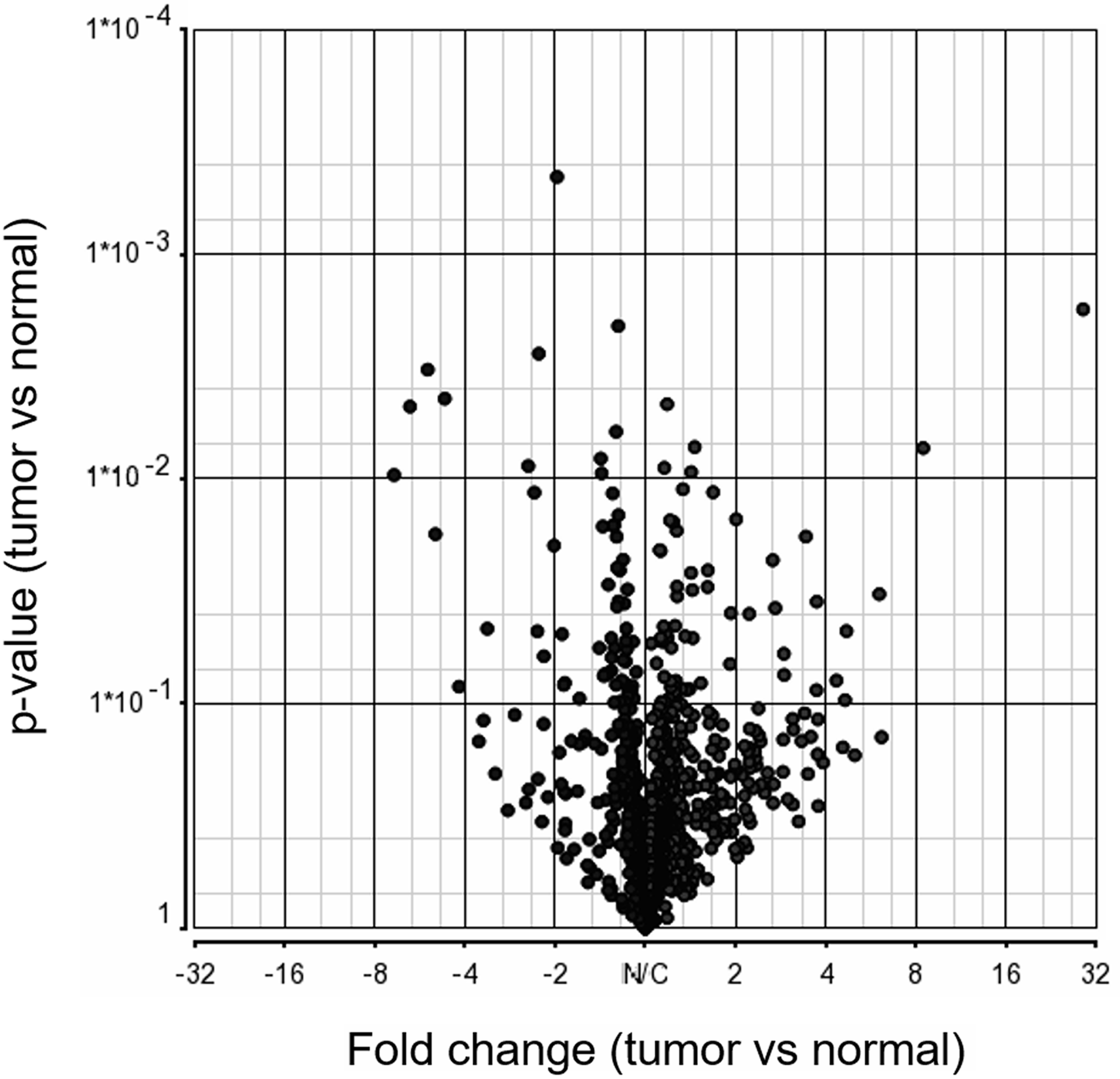

Supplementary Figure 1: A volcano plot represents the differentially expressed miRNAs in urinary EVs.

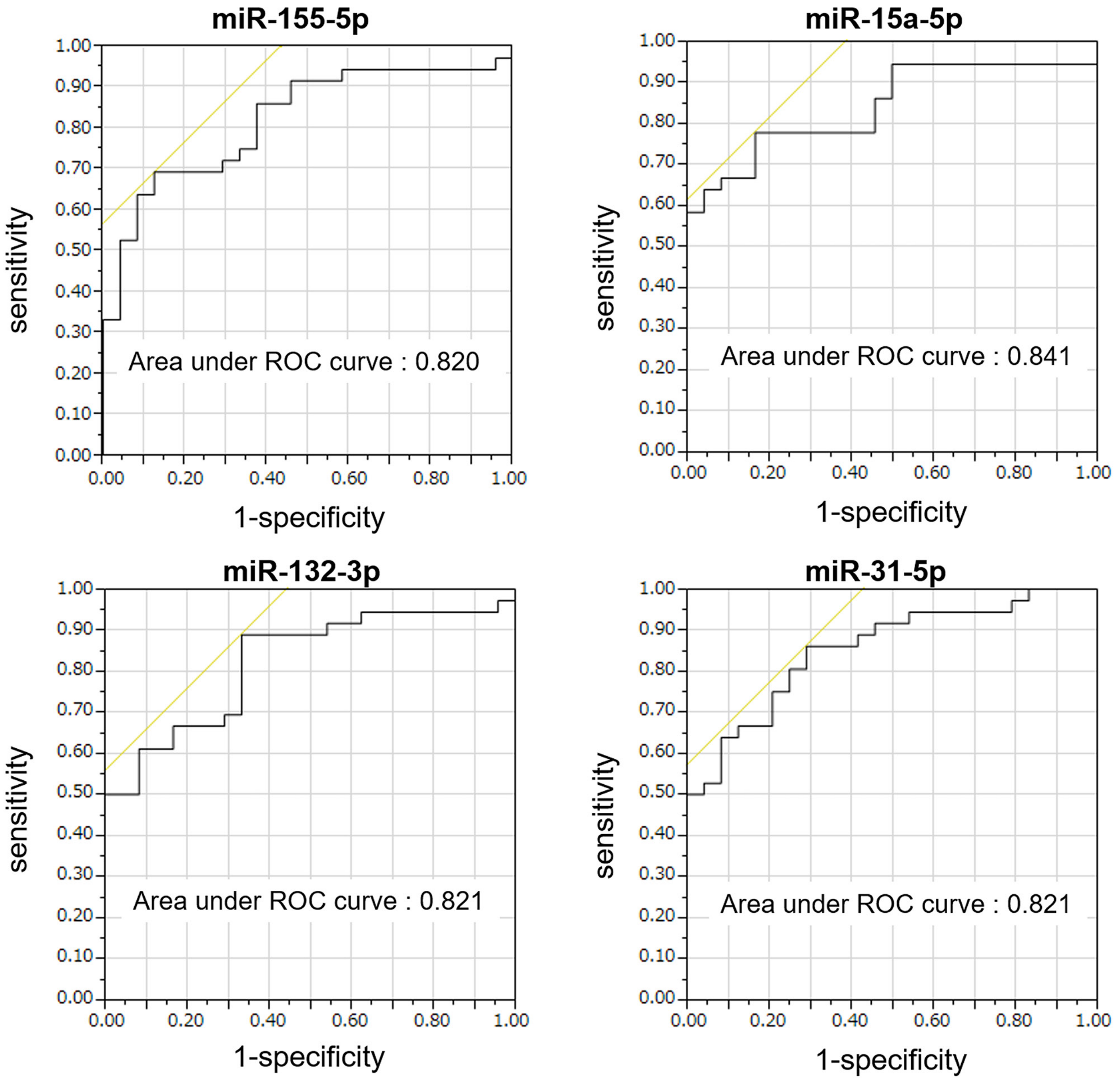

Supplementary Figure 2: ROC curve analysis using other 4 miRNAs in urinary EVs (miR-155-5p, miR-15a-5p, miR-132-3p and miR-31-5p).

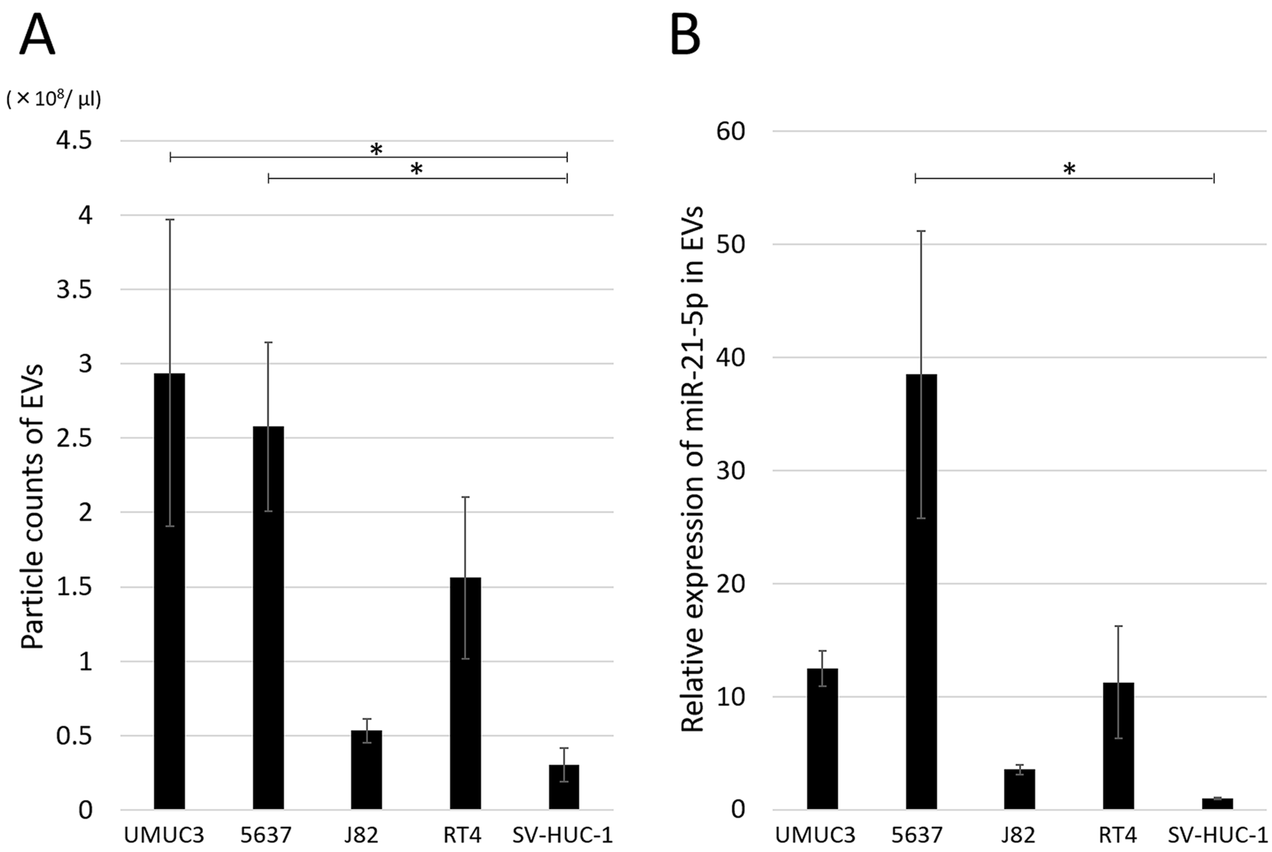

Supplementary Figure 3: A. The counts of EVs secreted from each cell lines (UMUC3, 5637, J82, RT4 and SV-HUC-1). **B.** The expression level of miR-21-5p in EVs secreted from each cell lines. Data are mean ± S.E. of 3 independent experiments. *$p < 0.05$.
